# Maximal aerobic speed: methodological implications and unintended consequences

**DOI:** 10.1007/s00421-026-06331-5

**Published:** 2026-07-30

**Authors:** Fernando Gabe Beltrami, Øyvind Støren, Jan Helgerud

**Affiliations:** 1 Department of Health Sciences and Technology, Exercise Physiology Lab, Gloriastrasse 37/39, 8092 Zurich, Switzerland; 2 Department of Sports, Physical Education and Outdoor Studies, University of South-Eastern Norway, Bø, 3800 Norway; 3 Medical Rehabilitation Clinic, Treningsklinikken, Trondheim, 7047 Norway

## Abstract

Maximal aerobic speed (MAS) represents the minimal speed where a further increase in speed will not result in a further increase in oxygen consumption (V̇O_2_). MAS is determined from the relationship between maximal V̇O_2_ (V̇O_2max_) and running economy, the latter also expressed as the O_2_ cost of running. There is no standardized method for determination of MAS in the literature, but three approaches are more dominant: the first approach consists of determining a loss of linearity between V̇O_2_ and speed. The second approach performs a linear extrapolation from the relationship between submaximal V̇O_2_ and speed towards V̇O_2max_. The third approach divides V̇O_2max_ by the O_2_ cost of running measured over one or more speeds. We explore the assumptions and flaws of the different approaches. We show that the first approach lacks sufficient objectivity. The second approach, based on linear extrapolation, is prone to significant error, particularly when changes in O_2_ cost happen at the lower or higher speeds tested, to the extent that higher sub-maximal V̇O_2_ for a given speed results in improved MAS and vice-versa. We suggest that MAS should be assessed using a simple division of V̇O_2max_ by the O_2_ cost of running determined from a race-relevant speed, which can be easily integrated into a single testing session.

## Introduction

Maximal aerobic speed (MAS) represents the minimal speed where a further increase in speed will not result in an increase in oxygen consumption (V̇O_2_), or, in other words, the minimal speed required to achieve maximal V̇O_2_ (V̇O_2max_) (Lacour et al. 1991). Although in this article MAS is discussed within the context of running, the concept can be readily used in other situations, such as skiing (Støren et al. 2023) or cycling (Støren et al. 2014), using power instead of speed when appropriate. MAS is determined from the relationship between V̇O_2max_ and running economy, the latter calculated either as V̇O_2_ at sub-maximal running speeds or converted into the O_2_ cost of running (C_r_, in ml kg^− 1^km^− 1^) by dividing V̇O_2_ by speed, paying attention to the appropriate conversion of the units of distance and time. MAS, therefore, has no regards to the actual capacity to maintain that speed or power for prolonged periods or the capacity to sustain steady-state, as is the case for variables such as critical speed or maximal lactate steady state (Nixon et al. 2021).

The concept of MAS nonetheless has relevance for endurance events (Morgan et al. 1989; Jones 1998), as it integrates the two main determinants of performance, the third one being the fraction of V̇O_2max_ sustained during an event, although the utility of the latter for differentiating athletes of different levels has been questioned recently, because athletes of different levels show a similar percentage of V̇O_2max_ at the lactate threshold (Johansen et al. 2025). It has also been argued that the fractional utilization of V̇O_2max_ is a result of the event time (and therefore performance), and not a determinant of it (Støa et al. 2010). Theoretically, if two athletes have the same MAS the one who runs an event at a higher percentage of their MAS will have a higher overall speed, but this can only be assessed retrospectively, and the percentage of V̇O_2max_ at the lactate threshold does not seem suited for discriminating the faster between these two athletes (Johansen et al. 2025).

Another benefit of the quantification of MAS is that it bypasses the issue of how to best express V̇O_2max_ or running economy, whether using absolute values, relative to body mass or the usage of different exponents in allometric scaling (Berg 2003; Helgerud et al. 2010). As long as the same denotation is used for both V̇O_2max_ and the submaximal VO_2_, the resulting MAS will be the same, providing a metric which is expressed in the same units as performance itself, i.e., a speed. Besides using similar units, it is important that both V̇O_2max_ and C_r_ are calculated and expressed in a way that minimizes variability and improves the practical value of MAS, including that changes in the underlying components of MAS affect it in a predictable manner. MAS has been calculated in several ways (Thron et al. 2024), as also reviewed by Billat and Koralsztein (1996), and no standard mathematical definition of MAS exists in the literature. Here, we consider some methodological implications of the different approaches and show how the choice of calculation could affect the results obtained on a similar dataset. We finish with a recommendation for standardizing the assessment of MAS.

### First approach – vV̇O_2max_

Billat and colleagues (1995) defined vV̇O_2max_ as “the lowest speed which elicited a V̇O_2_ value equal to VO_2max_. Subsequently, Billat and colleagues (1996) and Hanon and colleagues (2007) defined vV̇O_2max_ as “the first stage during an incremental test where V̇O_2max_ was attained (mean of the 3 consecutive and highest values) and where the subsequent increase in V̇O_2_ was less than 2.1 ml kg^− 1^ min^− 1^ for an increase in speed of 1 km h^− 1^ (the original protocol employed 3-min stages and 1 km h^− 1^ increase in speed between stages when approaching V̇O_2max_) (Billat et al. 1995). Moreover, V̇O_2max_ must be associated with a blood lactate, a respiratory exchange ratio and a heart rate equals respectively, to 8 to 12 mmol.l^− 1^, 1.1 and minimum 90% of the theoretical maximal HR (220-age)”. While both these definitions are particularly prone to small fluctuations in data within an exercise test (Myers and Ashley 1997; Beltrami et al. 2014), the latter definition is particularly problematic. If it is accepted that the O_2_ cost of running is independent of speed for a given runner, as has been repeatedly demonstrated (Brueckner et al. 1991; Di Prampero et al. 1993; Fletcher et al. 2009), then for an athlete with an O_2_ cost of running of 220 ml kg^−1^km^− 1^ the expected increase in V̇O_2_ for a 1 km h^− 1^ change in speed is 3.7 ml kg^−1^ min^− 1^. However, if the O_2_ cost of running is 170 ml kg^−1^ km^− 1^, within what is reported in high-level athletes (Jones et al. 2021), the expected increase in V̇O_2_ for a 1.0 km h^− 1^ change in speed will be only 2.6 ml kg^−1^ min^− 1^. Clearly, using an absolute 2.1 ml kg^−1^ min^− 1^ threshold for defining loss of linearity in V̇O_2_ increases does not work universally.

Although the work of Taylor and colleagues (1955) is used by Billat et al. (1995) to justify the fixed threshold of 2.1 ml kg^− 1^ min^− 1^ (Billat and Koralsztein 1996) to define the attainment of V̇O_2max_, Taylor et al. based this number on the fact that the average V̇O_2_ change between stages for their protocol (which was an increase in treadmill incline of 2.5% at each stage, not 1.0 km h^−1^ change in speed) was 4.2 ml kg^−1^ min^−1^. The authors then decided that differences in V̇O_2_ of less than half the expected values could be reasonably expected to represent a physiological plateau. Thus, a correct interpretation of that work would be to define V̇O_2max_ as the minimal speed where changes in V̇O_2_ are less than half of what would be expected (Beltrami et al. 2014). This approach, however, is also problematic during continuous tests, as multiple stages can have successive differences which would satisfy this criterion (Beltrami et al. 2014). The utilization of secondary criteria based on respiratory exchange ratio, heart rate or blood lactate levels does little to ensure the attainment of V̇O_2max,_ as these can both be attained long before V̇O_2max_ (Poole et al. 2008), or not achieved at all despite maximal efforts. Given the limitations of the method, we consider this approach to determining MAS less objective and more error prone than the other two.

Lastly, for avoidance of confusion within the literature, we note that both Daniels and Danies (1992) and Morgan and colleagues (1989) used the term speed at V̇O_2max_ and the abbreviation vV̇O_2max_ in their work. These authors, however, defined and calculated vV̇O_2max_ as described in our second approach (see section below), which is now termed MAS. Therefore, the vV̇O_2max_ of Daniels and Danies (1992) and Morgan et al. (1989) is not equivalent and should not confused with the more recent definition of vV̇O_2max_ used by Billat and colleagues (1996).

### Second approach

**Fig. 1:**
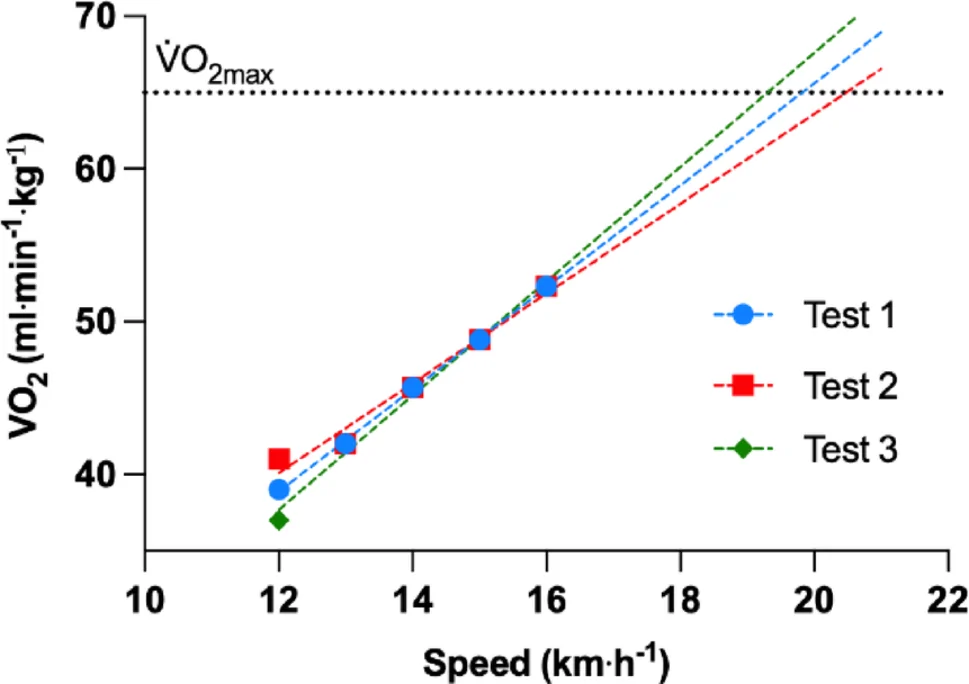
Three (theoretical) tests performed on the same athlete, with equal V̇O_2max,_ of 65.0 ml kg^−1^ min^−1^ in all three occasions, demarked by the dotted black line. Note how a change in V̇O_2_ at sub-maximal speeds has a large effect on the resulting MAS. By the nature of linear regressions, changes in sub-maximal V̇O_2_ at either extreme of the line will have larger effects on MAS. Changes in V̇O_2_ in the slower speeds, as seen in the example, will have contradictory effects on MAS: a higher submaximal V̇O_2_ will result in higher MAS, and vice-versa

The second approach to determining MAS is based on the extrapolation of the relationship between sub-maximal, steady-state V̇O_2_ and running speed to the value of the measured V̇O_2max_ (blue line in Fig. 1). Here, whether V̇O_2_ is expressed absolute, relative to body mass or using allometric scaling (Di Prampero et al. 1993; Støren et al. 2012; Rønnestad et al. 2020), is of no consequence to the calculated MAS. While this method is popular in the literature, it is particularly prone to large oscillations in MAS in response to small changes in sub-maximal V̇O_2_, whether these are biological or errors in measurement. Consider the three theoretical incremental tests with steady-state V̇O_2_ measurements during sub-maximal states from the same athlete who achieves a V̇O_2max,_ of 65 ml kg^− 1^ min^− 1^ in all three attempts of the test, shown in Fig. 1; Table 1. Using this method, an increase in V̇O_2_ during the first stage of the test (2.0 ml kg^−1^ min^− 1^ higher, implying a worse running economy at this stage) will improve MAS by 3.5% (red line in Fig. 1). This is because the effect of an increase in V̇O_2_ at the early parts of the extrapolation will be to flatten the relationship between V̇O_2_ and speed. The opposite will happen if V̇O_2_ decreases during the first stage (green line in Fig. 1): MAS will decrease. Although a lower V̇O_2_ would imply better running economy for that particular speed, it increases the steepness of the relationship between speed and V̇O_2_. Given the nature of linear interpolations, both the magnitude and direction of a given change in V̇O_2_ depends on where in the line the change occurs, as shown in Table 2. It can be seen that a similar change (in the example of Table 2 an increase in submaximal V̇O_2_ of 2.0 ml kg^− 1^ min^− 1^) will have an effect ranging from + 3.5 to −3.8% on the estimated MAS. The magnitude of the effect will be more pronounced the further the data is from the middle in either direction, as the linear regression is more sensitive to these data points. While C_r_ typically does not change between speeds at group level (Di Prampero et al. 1993; Fletcher et al. 2009; Jones et al. 2021), substantial within individual variability can be present between stages of a test. This is well illustrated by Fig. 2 in Jones et al. (2021), where C_r_ oscillated at times by more than 10.0 ml kg^− 1^ km^− 1^ in world-class runners between the different speeds of a single test. Such oscillations, whether biological or technical, can cause substantial variability in tests responses over time and mask or enhance true changes in MAS.

**Table 1:** Physiological data from Fig. 1

Speed (km h^− 1^)	Test 1		Test 2		Test 3	
	V̇O_2_ (ml kg^−1^ min^−1^)	C (ml kg^− 1^ km^− 1^)	V̇O_2_ (ml kg^−1^ min^−1^)	C (ml kg^− 1^ km^− 1^)	V̇O_2_ (ml kg^−1^ min^−1^)	C (ml kg^− 1^ km^− 1^)
12	**39.0**	**195**	**41.0**	**205**	**37.0**	**185**
13	42.0	194	42.0	194	42.0	194
14	45.7	196	45.7	196	45.7	196
15	48.8	195	48.8	195	48.8	195
16	52.3	196	52.3	196	52.3	196
V̇O_2max_ (ml kg^−1^ min^−1^)	65.0		65.0		65.0	
MAS (Method 2, in km h^− 1^)	**19.8**		**20.5**		**19.3**	
C_r_ (average all speeds) (ml kg^− 1^ km^− 1^)	**195.2**		**197.2**		**193.2**	
MAS (Method 3, in km h^− 1^)	**20.0**		**19.8**		**20.2**	

Values for V̇O2max in ml･kg^-1^･min^-1^ need to be multiplied by 60 before division by Cr in ml･kg^-1^･km^-1^ to obtain a speed in km/h

*MAS* maximal aerobic speed, *V̇O*_2_ oxygen uptake, *V̇O*_2max_ maximal oxygen uptake, *Cr* O_2_ cost. LInes highlighted in bold show different values between the different tests.

**Table 2:** Effects of changes in V̇O_2_ and VO_2max_ on MAS

Speed (km h^− 1^)	V̇O_2_ (ml·kg^−1^·min^−1^)					
	Baseline case	Change stage 1	Change stage 2	Change stage 3	Change stage 4	Change stage 5
12.0	39.0	**41.0**	39.0	39.0	39.0	39.0
13.0	42.0	42.0	**44.0**	42.0	42.0	42.0
14.0	45.7	45.7	45.7	**47.7**	45.7	45.7
15.0	48.8	48.8	48.8	48.8	**50.8**	48.8
16.0	52.3	52.3	52.3	52.3	52.3	**54.3**

	Delta MAS for a 5% increase in V̇O_2max_ (from 65.0 to 68.2 ml · kg^−1^· min^−1^)					
MAS (method 2, %)	+ 4.9%	+ 5.4%	+ 5.2%	+ 5.0%	+ 4.7%	+ 4.6%
MAS (method 3, %)	+ 5.0%	+ 5.0%	+ 5.0%	+ 5.0%	+ 5.0%	+ 5.0%

V̇O_2max_ (ml kg^−1^ min^−1^)	65	65	65	65	65	65
MAS (method 2, km h^− 1^)	19.8	20.5 (+ 3.3%)	20.1 (+ 1.2%)	19.7 (−0.6%)	19.4 (−2.3%)	19.1 (−3.8%)
MAS (method 3, km h^− 1^)	20.0	19.8 (−1.0%)	19.8 (−1.0%)	19.8 (−1.0%)	19.8 (−1.0%)	19.8 (−1.0%)

The different cases at the top present a baseline case and the effect of a change in sub-maximal V̇O_2_ of + 2.0 ml･kg^-1^･min^-1^ at each of the different sub-maximal stages according to method 2 and 3 for determination of MAS. Values in bold are different from baseline case. The bottom of the table shows the relative delta (%) in MAS resulting from a 5% increase in V̇O_2max_ in each case. As the different cases have different slopes, the changes in V̇O_2max_ will vary the magnitude of change in MAS when method 2 is used. This is not the case with method 3, where the changes in V̇O_2max_ are equal in direction and magnitude to the changes in V̇O_2max_, as no linear interpolation is used

*MAS* maximal aerobic speed, *V̇O*_2_ oxygen uptake, *V̇O*_2max_ maximal oxygen uptake

A similar issue is present with regards to variations in V̇O_2max_. Because the slope of the V̇O_2_ x speed relationship is not necessarily 1.0, a given change in VO_2max_ will have a smaller (if the slope is higher than 1.0) or larger (if the slope is lower than 1.0) effect on the calculated MAS. An example of this situation is shown in bottom of Table 2, where it can be seen that a 5% increase in V̇O_2max_ will increase MAS by between 4.6 and 5.4% depending on the slope of the V̇O_2_ x speed relationship.

### Third approach

Instead of relying on a linear extrapolation of submaximal V̇O_2_ to VO_2max_, the third approach divides V̇O_2max_ by C_r_ (Di Prampero et al. 1993; Rønnestad et al. 2020) to obtain a speed. Care must be taken to use the appropriate units: V̇O_2max_ in ml kg^−1^ min^−1^ needs to be multiplied by 60 to yield V̇O_2max_ in ml kg^− 1^ h^− 1^ before a division by C_r_ results in a speed in km h^− 1^. Here, both C_r_ and V̇O_2max_ can also be calculated gross (i.e. including) or net (i.e. deducting) of the resting V̇O_2_ (Brueckner et al. 1991). Using the same data as in Fig. 1, the average C_r_ across the tested sub-maximal speeds and the resulting MAS calculated using method 3 is shown in Table 1 (bottom two lines). Here, the 2.0 ml kg^− 1^ min^− 1^ change in O_2_ cost during the first stage of the test only results in a 1% increase in C_r_, since the submaximal V̇O_2_ was constant for the other four speeds. Accordingly, the calculated MAS only varies by 1%, in the same direction and magnitude as the changes in C_r_. This approach also makes the resulting MAS independent of where a change in C_R_ occurs within the range of speeds selected, whether of biological or technical nature. As shown in Table 2, an increase in submaximal V̇O_2_ of 2.0 ml kg^−1^ min^− 1^ in any of the speeds used to determine C_r_ would have a similar effect on MAS (a decrease of 1.0%), whereas the magnitude of the effect ranged between + 3.5 – −3.8% with the linear interpolation method. Likewise, any changes in V̇O_2max_ (Table 2) will have an effect which is similar in direction and magnitude in the calculated MAS.

One source of confusion with this method might be which stages are to be used when determining C_r_. Although C_r_ is stable over a range of speeds (Helgerud et al. 2010), this needs to be standardized so that researchers and practitioners do not fall into the trap of using different numbers at every new measurement for a given athlete, or go outside the range where the relationship between V̇O_2_ and speed is linear. This issue is addressed in the final section of the manuscript (Practical recommendations).

## Discussion

We examined the implications and limitations of different methodologies currently available to determine MAS during running in athletes. The first approach, based on the minimal speed where V̇O_2max_ is reached, lacks sufficient accuracy in the quantification and should not be recommended. The second approach, based on linear extrapolation of the V̇O_2_ x speed relationship, is prone to unintended results, including that higher V̇O_2_ values in the early stages of a test can lead to higher MAS and vice-versa, a finding which is hard to justify from a physiological standpoint. Calculating MAS according to the third approach, by dividing V̇O_2max_ by C_r_ (determined from a single relevant speed or averaged from a range of relevant speeds) retains the desirable properties of the calculation, integrating running economy and V̇O_2max_ into a single value. Values then only change in the expected direction, i.e., MAS will decrease if either V̇O_2max_ decreases or C_r_ increases, or vice-versa. Moreover, the magnitude of the changes will be directly proportional to the changes in both V̇O_2max_ and C_r_, with no speed exerting greater impact on the results, in lines with the notion that C_r_ is constant across speeds. Central to both methods 2 and 3, evidently, is the need to collect appropriate values of sub-maximal V̇O_2_ or C_r,_ paying attention that the data is collected at speeds where true steady-state can be achieved in both V̇O_2_ and CO_2_ output (Gaesser and Poole 1996). While there are also arguments in the literature about whether running economy or C_r_ should be determined using V̇O_2_ or metabolic power (in Joules) (Fletcher et al. 2009; Shaw et al. 2014), this discussion is not relevant to the current work, as the difference between the calculation methods remains the same.

Rejection of the slope-based estimation of MAS does not mean that different athletes cannot have different V̇O_2_ vs. speed slopes. Different slopes are in fact expected for athletes with different C_r_. Daniels and Daniels (1992) had already noted that athletes with different specializations showed different slopes in their V̇O_2_ x speed relationship, with middle distance athletes showing flatter slopes compared with long distance athletes. They put forward the idea that running economy adapts better for the speed that is most relevant to the athlete. We note, however, that no subsequent study attempted to validate these findings, which come from a small set of well-trained individuals. Regardless of whether training can or cannot change the slope of V̇O_2_ x speed in runners, determination of MAS via the extrapolation method will be exposed to undesirable variation. When using low, non-relevant speeds in the extrapolation, these might have a disproportional influence in the calculation of MAS. An example of this can found in the work of Jones (1998), following an Olympic runner over 5 years: the MAS of the athlete (calculated using linear interpolation of V̇O_2_ from speeds between 14.0 and 19.0 km h^− 1^) increased by 2.6% between 1992 and 1993 (from 19.5 to 20.0 km h^− 1^). This happened even though V̇O_2max_ decreased by 3.6% (from 68.5 to 66.0 ml kg^− 1^ min^− 1^). Running economy, reported only at 16.0 km h^− 1^, improved by 3.8% (from 53.0 to 51.0 ml kg^− 1^ min^− 1^), thus barely enough to offset the drop in V̇O_2max_. Therefore, the only explanation for the increase in MAS is that the slope of the V̇O_2_ x speed relationship must have flattened significantly, which could only have happened if either V̇O_2_ increased at the lower sub-maximal speeds used (which would be interpreted as a worse running economy at those speeds) or if it decreased at the higher speeds used, or a combination of both. Deriving MAS using method 3, from the race-relevant speeds, could have provided a clearer picture of the mechanisms behind the changes in MAS, or indeed have given an altogether different MAS value, as in the example from Table 1.

### Practical recommendation

One possible solution for robust determination of MAS is to use only a range of speeds which are relevant to the population at hand. If an athlete competes at speeds ranging between 18.0 and 20.0 km h^− 1^, what is the value of allowing the variations in C_r_ at much lower speeds, for example 14.0 km h^− 1^, influence the resulting MAS? It could be argued that since endurance athletes have a speed range of 2.0–3.0 km h^− 1^ from 5-km races to the marathon, depending on their level, this speed range should be used for determination of MAS, thus decoupling it from lower, less relevant speeds from an incremental test. C_r_ has been shown to be stable over a range of speeds between 70 and 90% of V̇O_2max_ (Helgerud et al. 2010), and this represents speeds typically encountered during endurance events. If this range of speed is within that of endurance performances, the prerequisite of achieving steady-state will also be met, although this needs checking for each athlete at every speed tested.

Given that MAS is commonly measured in athletes or endurance-trained individuals, for whom performance level is known or can be reasonably estimated, we propose that quantification of MAS for research studies can be performed based on a speed expected to yield approximately 75% of V̇O_2max_ for athletes in endurance events lasting 2 or more hours, based on expected marathon speed, and close to 90% of V̇O_2max_ for athletes focusing on shorter events. For the assessment of individual athletes, it makes most sense to simply use the speed which is more race relevant. In either case, this assessment can be easily integrated into a single day testing session where a preliminary warm-up is followed by 5-min bouts at the target speed, which is then followed by a ramp test to determine V̇O_2max_. To improve the accuracy of measures, the speeds used for determination of C_r_ should be tested at least in duplicate and results averaged together.
